# The Contribution of Evolutionary Game Theory to Understanding and Treating Cancer

**DOI:** 10.1007/s13235-021-00397-w

**Published:** 2021-08-30

**Authors:** Benjamin Wölfl, Hedy te Rietmole, Monica Salvioli, Artem Kaznatcheev, Frank Thuijsman, Joel S. Brown, Boudewijn Burgering, Kateřina Staňková

**Affiliations:** 1 Department of Mathematics, University of Vienna, Vienna, Austria; 2 Vienna Graduate School of Population Genetics, Vienna, Austria; 3 Department of Molecular Cancer Research, University Medical Center Utrecht, Utrecht, The Netherlands; 4 Department of Mathematics, University of Trento, Trento, Italy; 5 Department of Data Science and Knowledge Engineering, Maastricht University, Maastricht, The Netherlands; 6 Department of Biology, University of Pennsylvania, Philadelphia, USA; 7 Department of Computer Science, University of Oxford, Oxford, UK; 8 Department of Integrated Mathematical Oncology, H. Lee Moffitt Cancer Center and Research Institute, Tampa, FL USA; 9 Department of Biological Sciences, University of Illinois at Chicago, Chicago, IL USA; 10 The Oncode Institute, Utrecht, The Netherlands; 11 Department of Engineering Systems and Services, Faculty of Technology, Policy and Management, Delft University of Technology, Delft, The Netherlands

**Keywords:** Evolutionary game theory, Eco-evolutionary dynamics, Stackelberg evolutionary games, Competitive release, Resistance, Genetics, 91A22, 91A65, 91A80, 92D25, 92D40

## Abstract

Evolutionary game theory mathematically conceptualizes and analyzes biological interactions where one’s fitness not only depends on one’s own traits, but also on the traits of others. Typically, the individuals are not overtly rational and do not select, but rather inherit their traits. Cancer can be framed as such an evolutionary game, as it is composed of cells of heterogeneous types undergoing frequency-dependent selection. In this article, we first summarize existing works where evolutionary game theory has been employed in modeling cancer and improving its treatment. Some of these game-theoretic models suggest how one could anticipate and steer cancer’s eco-evolutionary dynamics into states more desirable for the patient via evolutionary therapies. Such therapies offer great promise for increasing patient survival and decreasing drug toxicity, as demonstrated by some recent studies and clinical trials. We discuss clinical relevance of the existing game-theoretic models of cancer and its treatment, and opportunities for future applications. Moreover, we discuss the developments in cancer biology that are needed to better utilize the full potential of game-theoretic models. Ultimately, we demonstrate that viewing tumors with evolutionary game theory has medically useful implications that can inform and create a lockstep between empirical findings and mathematical modeling. We suggest that cancer progression is an evolutionary competition between different cell types and therefore needs to be viewed as an evolutionary game.

## Introduction

Cancer is a disease of unregulated proliferation, caused by abnormal function of genes responsible for regulating cell division. The genesis of cancer has strong ties to human life history [6, 70, 93, 94, 179], and its progression is driven by natural selection, characterized by cancer cells exhibiting the following three conditions [58]:

1. The presence of heritable variation: Heritable traits vary among different cancer cells, ultimately as a result of genetic mutations, epigenetics, chromosomal re-arrangements and other mechanisms associated with genetic instability.
2. A struggle for existence: There are limits to growth due to competition for limited space and resources.
3. The influence of heritable variation on the struggle for existence: Generally, the likelihood of cell survival depends on its own traits, and the traits of the others. Cells with traits that confer higher chances of survival and proliferation will in time increase in frequency (frequency-dependent selection) [93, 94].

This Darwinian view of cancer aligns with the premises of evolutionary game theory (EGT), which assumes that evolution tests heritable traits in an ongoing competition for survival [38, 100, 127, 128]. EGT is a branch of mathematics that has helped to conceptualize and understand the behavior of real-world biological systems, including several counter-intuitive biological phenomena [91, 92, 128, 162, 182, 201], and is being increasingly recognized as an important tool for mathematical oncologists [18, 154, 165].

EGT deals with situations where organisms using different strategies and/or possessing different traits interact with each other. Unlike in classical game theory [139, 190, 191], these organisms do not need to be overtly rational, i.e., their strategies (often referred to as “types”) are inherited rather than rationally chosen [37, 100] (although a rational population-level interpretation of the dynamics is also possible [109]). Some strategies might confer higher fitness and the individuals using these strategies will in the long run dominate the population. Thus, if we see cancer as a Darwinian process, it can be described as an evolutionary game, where cancer cells are the players, their heritable traits correspond to the strategies, and the payoffs are represented in terms of survival and proliferation (fitness) [38, 129]. This is a dynamic game, as one can analyze how frequencies of different strategies and numbers of individuals corresponding to these different strategies change in time. We refer to those changes as evolutionary and ecological dynamics, respectively. Both together are called eco-evolutionary dynamics.

Compared to other fields of applied mathematics, EGT of cancer is a relatively new field, just a few decades old [123, 181]. Tomlinson was first to explicitly frame cancer as an evolutionary game [181]. Since then, at least 60 publications on cancer have called their research game-theoretic. This body of literature has grown into diverse and different groupings. Given that cancer is an evolutionary process, cancer treatment could benefit from insights from evolutionary theory, giving rise to Evolutionary or Darwinian medicine [77, 79, 83]. The increasing interest in this field is reflected in the recent update of medical curricula to include evolutionary reasoning [140]. Clearly, EGT can only improve cancer treatment if there is something gained from these evolutionary insights. Standard of Care (SoC) in treating cancer typically applies therapy at Maximum Tolerable Dose (MTD), to remove as many tumor cells as fast as possible. For some aggressive cancers, such as advanced Non-Small Cell Lung Cancer, no better treatment than MTD has been found so far [9, 19, 20]. Yet, unless the patient is cured, the MTD strategy promotes evolution of treatment-induced resistance which leads to treatment failure [76, 164, 206].

The fact that even personalized therapies tailored to the cancer’s genetic signature and to the individual’s genetic disposition fail can be attributed to the extensive adaptive potential of the human genome. As MTD can only eradicate therapy-sensitive tumor cells, it benefits therapy-resistant cells [82, 146]. Subsequently, growth-limiting constraints due to competition may temporarily vanish and increase the *per capita* growth rate of the resistant types (competitive release [50, 68, 204]). In turn, some experiments show that when treatment is stalled (drug holiday), resistant types are typically at a disadvantage (cost of resistance [168], although this is not universal [113]). This evidence suggests that MTD might be evolutionarily unwise when it promotes treatment-induced resistance in cancer cells. Additionally, there is evidence for selection for evolvability in tumor cells, e.g., hyper-mutators [44]. Recent works showed that a game-theoretic approach may help to provide an alternative to MTD, based on anticipating and steering the cancer eco-evolutionary dynamics in response to the treatment [88, 165].

The aims of this paper are: (1) to discuss the achievements of the existing works on game theory of cancer and (2) to show the future potential of game theory to understand cancer mechanisms, inspire novel research, and design better treatment protocols.

In the remainder of this paper, we first introduce models where the interaction among cells is explicitly framed as an evolutionary game, with either no or fixed treatment (Sect. 2). Second, we will review cancer models where the physician, as a rational player optimizing their own objective(s), enters the evolutionary game (Sect. 3). Third, we will focus on the clinical aspects of EGT therapy models (Sect. 4). We close with a discussion on limitations and future steps in game theory of cancer and its treatment (Sect. 5).

### Mathematical Background

Cancer is a Darwinian disease, in which cancer cells play an evolutionary game between each other within the dynamic environment of the tumor that also includes diverse normal cells (stroma) [21, 84, 181]. The cells may have different types, varying in their (possibly evolving) level of resistance to a particular treatment or treatment combination [77, 84, 124, 164]. Here, we do not specify whether these types are just phenotypically or also genetically different—that is why we confine ourselves to the term “types”, as opposed to “clones” used in some literature. For some cancers, such as metastatic Castrate-Resistant Prostate Cancer (mCRPC) and Estrogen Receptor Positive (ER+) breast cancer, cancer types differing in their resistance levels with respect to a particular treatment have been identified both in vitro and in vivo [67, 73, 83, 207]. For less researched cancers, such types have not been established yet and it may be that the level of resistance varies per cancer cell and/or evolves in response to treatment [151].

Therefore, in the most general game-theoretic model of cancer, the resistance of a particular cancer cell type to a particular treatment is a continuous evolving heritable trait. Then, individual cancer cells are identified by their value of this trait, which is subject to natural selection. Here, we will adopt the *Darwinian dynamics* approach to describe such a situation, expanding the original model of Vincent and Brown into more dimensions [186].

A vector $\mathbf{x }(t)=(x_1(t),\ldots ,x_n(t))^T$ defines population densities (population size) of cancer cells of types ${\mathcal {T}}=\{1,\ldots , n\}$ at time *t*. The fitness of cancer cells of type $i\in {\mathcal {T}}$ may depend on the densities and traits of all cancer cell types. Consequently, the ecological dynamics of cancer cells of type *i* are given by

1 $$\begin{aligned} \frac{\mathrm{d}x_i(t)}{\mathrm{d}t}&=x_i(t)\cdot H_i ({\mathbf {U}}(t),{\mathbf {x}}(t),{\mathbf {m}}(t)) . \end{aligned}$$

Here, ${\mathbf {U}}(t)=\left( u_{ij}(t)\right)$ is a resistance matrix, where $u_{ij}(t)\in [0,1]$ indicates the resistance level of cancer cells of type *i*, in response to treatment $j \in \Theta =\{1,\ldots ,p\}.$ Moreover, ${\mathbf {m}}(t)=(m_1(t),\ldots , m_p(t))^T$ is the vector of doses for each therapy option from the treatment set $\Theta .$ Without loss of generality, we can assume that $m_j(t)\in [0,1]$ for all $j\in \Theta ,$ where $m_j(t)=1$ and $m_j(t)=0$ correspond to the MTD and no dose of treatment *j* at time *t*, respectively. In this formulation, we see that the *per capita* growth rate $H_i({\mathbf {U}}(t),{\mathbf {x}}(t),{\mathbf {m}}(t))$ of type *i* may give rise to both density and frequency-dependent dynamics, as it depends on ${\mathbf {x}}(t)$ explicitly.

Vincent and Brown developed the concept of a *fitness generating function* (G-function) as a way to describe the fitness of many species (or types) by making use of a single mathematical expression [186]. A function $G(\mathbf {v},{\mathbf {U}}(t),{\mathbf {x}}(t),{\mathbf {m}}(t))$ is said to be a fitness generating function (G-function) of the population dynamics (1) if

2 $$\begin{aligned} \left. G\left( {\mathbf {v}}(t),{\mathbf {U}}(t),{\mathbf {x}}(t),{\mathbf {m}}(t)\right) \right| _{{\mathbf {v}}(t)=\left( u_{i1}(t),\ldots ,u_{ip}(t)\right) }=H_i({\mathbf {U}}(t),{\mathbf {x}}(t),{\mathbf {m}}(t)), \end{aligned}$$

where ${\mathbf {v}}$ is a virtual vector variable. Replacing $v_j$ in the G-function with $u_{ij}$ for each $j\in \Theta$ yields the fitness of an individual cell of type *i* in a population defined by the same G-function. Using the G-function, we can rewrite Eq. (1) as

3 $$\begin{aligned} \frac{\mathrm{d}x_i(t)}{\mathrm{d}t}&=x_i(t)\cdot \left. G\left( {\mathbf {v}}(t),{\mathbf {U}}(t),{\mathbf {x}}(t),{\mathbf {m}}(t)\right) \right| _{{\mathbf {v}}(t)=\left( u_{i1}(t),\ldots ,u_{ip}(t)\right) }. \end{aligned}$$

Cancer types with a higher *per capita* growth rate will persist in the population. Therefore, the dynamics of the evolution of resistance $u_{ij}$ of the cancer cell of type *i* in response to a treatment *j* (evolutionary dynamics) are given as

4 $$\begin{aligned} \frac{\mathrm{d}{u}_{ij}(t)}{\mathrm{d}t}&=k_{ij}\, \frac{\partial H_i \left( {\mathbf {U}}(t),{\mathbf {x}}(t),{\mathbf {m}}(t)\right) }{\partial u_{ij}(t)}, \end{aligned}$$

which can be rewritten using the G-function as:

5 $$\begin{aligned} \frac{\mathrm{d}{u}_{ij}(t)}{\mathrm{d}t}&=k_{ij}\, \frac{\partial G \left( {\mathbf {v}}(t),{\mathbf {U}}(t),{\mathbf {x}}(t),{\mathbf {m}}(t)\right) }{\partial v_{j}(t)}\Bigr |_{{\mathbf {v}}(t)=\left( u_{i1}(t),\ldots ,u_{ip}(t)\right) }. \end{aligned}$$

Here, $k_{ij}$ is a speed parameter, which is a measure of heritability and additive genetic variance, in line with Fisher’s fundamental theorem of natural selection [69]. This speed parameter may be influenced by many other factors, like mutation rates, population size, population structure and the underlying genetics of inheritance. For example, in *adaptive dynamics*, $k_{ij}$ is linearly increasing with population size and stochastic with respect to other parameters (canonical equation of adaptive dynamics [61, 86, 95, 130]). For the sake of simplicity, when modeling (5), it is often assumed that $k_{ij}$ is the same constant for all *i* and *j*, while one could easily imagine that $k_{ij}$ varies in time and may be a (likely nonlinear) function of $x_{i}(t)$. In the remainder of this paper, we will not write out the time-dependence explicitly; thus, we shall use ${\mathbf {U}}$, ${\mathbf {x}}$ and ${\mathbf {m}}$ instead of ${\mathbf {U}}(t)$, ${\mathbf {x}}(t)$ and ${\mathbf {m}}(t)$, respectively. Equations (3) and (5) constitute the Darwinian dynamics, describing the ecological and evolutionary dynamics of cancer cells, respectively.

If the ecological dynamics (3) converge to a stable equilibrium ${\mathbf {x}}^*\ge 0$, we call ${\mathbf {x}}^*$ an *ecological equilibrium*. Each combination of resistance and treatment strategies $({\mathbf {U}}, {\mathbf {m}})$ may have an associated vector of stable population sizes ${\mathbf {x}}^*$, with ${\mathbf {x}}^*_i \ge 0 \quad \forall i \in \{1,2,\ldots ,n\}$. A generic ${\mathbf {U}}$ may have one or more values of ${\mathbf {x}}^*,$ or no equilibrium associated with it, depending on the G-function. Moreover, even if we assume that the ecological equilibrium exists for any choice of ${\mathbf {U}}$ and ${\mathbf {m}},$ it may be that only a subset of possible values of ${\mathbf {U}}$ and ${\mathbf {m}}$ will correspond to positive equilibrium population sizes. Depending upon the model, its parameters and the strategies ${\mathbf {U}}$ and ${\mathbf {m}}$, there will likely be an upper limit to the number of types that can co-exist at positive population sizes [90].

Solved together for ${\mathbf {m}}$ fixed at particular values, Eqs. (3) and (5) often determine an equilibrium solution for ${\mathbf {x}}({\mathbf {m}})$ and ${\mathbf {U}}({\mathbf {m}}),$ which we will denote by ${\mathbf {x}}^*({\mathbf {m}})$ and ${\mathbf {U}}^*({\mathbf {m}}),$ respectively. The nonzero equilibrium values of $x^*_i({\mathbf {m}})$ and their associated strategies $\left( u^*_{i1}({\mathbf {m}}),\ldots , u^*_{ip}({\mathbf {m}})\right)$ form a ‘coalition’ of strategies. If, for a particular choice of ${\mathbf {m}}$, these strategies resist invasion by other mutant strategies, they are called Evolutionarily Stable Strategies (ESSs) with respect to treatment ${\mathbf {m}}$ [100]. A necessary condition for an ESS is that the G-function maximizes *G* with respect to $\mathbf{v }$ at the corresponding ${\mathbf {x}}^*$. Further stability properties of the ESS can be analyzed (e.g., convergence stability or neighborhood invasion stability [10, 11]).

In Sect. 2, we will consider existing models of cancer *without treatment* and those that consider a *predefined fixed treatment*. Such treatments may administer a constant dose ${\mathbf {m}}$ or, for example, pause treatment when the total tumor population is below a certain predefined threshold, and re-administer it again once the population of tumor cells recover to its initial size. In Sect. 3, we will consider situations where the physician enters the ‘game against cancer’ as a rational player, i.e., a player optimizing certain objective(s) with respect to their treatment strategies, as opposed to executing an *a priori* decided treatment strategy.

## Game Theory of Cancer Without Treatment or with a Predefined Treatment Regimen

In the literature of EGT models of cancer with no or predefined treatment ${\mathbf {m}}$, the authors either focus on finding the ESS resistance strategy ${\mathbf {U}}^*$ at the ecological equilibrium ${\mathbf {x}}^*$, or they analyze transient dynamics toward $\left( {\mathbf {x}}^*,{\mathbf {U}}^*\right)$ for particular (predefined) choices of ${\mathbf {m}},$ to see what choices of ${\mathbf {m}}$ are better than others in terms of some prespecified metrics, such as progression-free or overall survival.

We will first present research that utilizes both Eqs. (3) and (5), followed by models that somewhat simplify the two equations by using a fitness and a competition matrix, respectively, and spatial models.

### Models with Eco-evolutionary Dynamics Described by Equations (3) and (5)

Reed et al. introduce the following G-function

6 $$\begin{aligned} G=r \left( (1-v_1)(1-v_2)(1-v_3)-\frac{x}{K}\right) -\mu _1(v_1)-\mu _2(v_2)-\mu _3(v_3) \end{aligned}$$

in their commentary on treating pediatric sarcomas [153]. Here, $v_i$ denotes the treatment-induced resistance to treatment $i\in \{1,2,3\}$, *r* is the intrinsic growth rate of the tumor cells, and $\mu _i(v_i)=\frac{m_i}{k_i+b_i v_i}$ is the treatment-induced death rate for treatment *i*. In $\mu _i(v_i)$, $m_i$ is the base treatment-induced death rate of the tumor cells, $k_i$ denotes innate resistance, and $b_i$ gives the benefit gained by accumulating resistance toward drug *i*. Reed et al. adopt the framework given by (3) and (5) with a G-function defined by (6) to analyze possible strategies to combat the pediatric sarcoma, motivated by the theory of extinction from ecology, recently discussed in the oncology literature as well [78, 85, 153].

Motivated by numerical simulations on different treatment regimens, the authors suggest that when a cure for pediatric sarcoma is an achievable outcome, the first strike (standard of care) therapy should be either augmented, or closely followed with diverse second strike therapies. They hypothesize that application of the “first-strike” and “second-strike” therapies may improve the standard of care, which typically relies on continuous MTD therapy with a drug or drug combination until disease progression (or unacceptable toxicity for the patient), or no therapy at all if the disease is in remission.

In contrast, when it is believed that a cure is unachievable, Reed et al. propose the adaptive therapy protocol used by Zhang et al. for metastatic castrate-resistant prostate cancer, which we will discuss in Sect. 2.5 [153, 206].

### Replicator Dynamics with Fitness Matrix

The simplest and often very intuitive game-theoretic cancer models are those where the fitness of cancer cells is given by a fitness matrix. These models typically assume that the cancer cells engage in pairwise interactions and, as a result of these interactions, the cells may reproduce, generating offspring of the same type as the parental cell (although other interpretations are also possible [109, 113]).

Let $a_{ij}$ be the expected number of offspring generated by a cancer cell of type *i* interacting with a cell of type *j*. Alternatively, if $a_{ij}\in [0,1],$ it can define the probability of a cell of type *i* producing an offspring of its own type when interacting with a cell of type *j*. If we have *n* types of cancer cells and we know $a_{ij}$ for all $i,j\in \{1,\ldots ,n\}$, we can construct an $n\times n$ fitness matrix ${\mathbf {A}}=(a_{ij})$. The population (ecological) dynamics of cells of different types as proportions, ${\mathbf {q}}$, instead of densities, ${\mathbf {x}}$, are commonly described by *replicator dynamics* [25, 26, 109, 113, 115, 172, 173, 194], where $q_i=\frac{x_i}{\sum _{i=1}^n x_i}$ is defined by

7 $$\begin{aligned} \frac{\mathrm{d}q_i}{\mathrm{d}t}&= q_i \left( ({\mathbf {A}}\,{\mathbf {q}})_i - {\mathbf {q}}^T\, {\mathbf {A}}\, {\mathbf {q}} \right) . \end{aligned}$$

Here, the *per capita* growth rate of cancer cells of type *i* is given by their expected payoff (fitness) $({\mathbf {A}}\,{\mathbf {q}})_i$ minus the mean fitness of the entire population ${\mathbf {q}}^T\, {\mathbf {A}}\, {\mathbf {q}}$. This fitness is frequency-dependent [49, 112] and captures non-cell-autonomous effects that are central to the ecology of cancer [65, 113, 125].

The replicator dynamics represent a special case of (3) and (5) as it considers population (ecological) dynamics only in terms of proportions and does not consider the evolutionary dynamics of different types of cancer cells. The latter point implies that it fits within the framework set by Eq. (3) with the G-function defined by $({\mathbf {A}}\,{\mathbf {q}})_i - {\mathbf {q}}^T\, {\mathbf {A}}\, {\mathbf {q}},$ where trait ${\mathbf {U}}$ simply does not evolve. The dynamics of frequencies $q_i$ with $i=1,\dots ,n$ are restricted to the *n*-dimensional simplex, i.e., $\sum _{i=1} ^{n} q_i=1.$

As shown by Zeeman, any ESS of matrix ${\mathbf {A}}$ is an attractor (stable equilibrium) of the replicator dynamics (7) [203]. If such an ESS in tumors exists, reaching it using available therapies could provide a means for achieving *long-term stabilization* of tumors and a significant increase in progression-free and overall survival [56, 113, 194]. However, it is important to be aware of the timescales involved and that the equilibria might not be reached [49, 111, 112], for example due to ecological constraints on population size [87].

One of the first models that defines the competitive interactions of cancer cells via a fitness matrix following (7) was called ‘Go-vs-Grow game’ as introduced by Basanta et al. [24]. This model was promptly extended to include glycolysis [26]. Here, the interaction between three cancer cell types of invasive (Go), autonomous growth (Grow) and glycolytic (GLY) types was introduced and it was analyzed for how different parameters in the fitness matrix ${\mathbf {A}}$ influence the game characteristics and ESSs [26]. The main outcomes of this analysis are that an invasive cancer type is more likely to evolve after the occurrence of the glycolytic type, and that the therapies increasing the fitness cost of switching to anaerobic glycolysis might decrease the probability of the emergence of a more invasive cancer type. The follow-up work includes stromal cells interacting with different types of cancer cells and their role in promoting cancer invasiveness [25]. Dingli et al. showed that targeting the interactions between the tumor and the stromal cells, so that the latter outcompete the former ones, can be a more promising approach, compared to targeting the cancer cells directly [64]. Other examples of cancer games with the fitness defined by a matrix are the cooperative ones, following the paper of Axelrod et al. summarizing evidence of cooperation among cancer cells [21].

### Estimating Parameters of the Fitness Matrix

Although the above works modeled the interactions between cancer cells of different types and their environment as a fitness matrix, the parameters of these matrices were not directly measured, neither in vivo nor in vitro. To remedy this, Kaznatcheev et al. introduced a technique to directly estimate parameters of the fitness matrix of replicator dynamics from data measured in vitro [113]. They studied interactions of different cancer cell types in co-cultures of non-small cell lung cancer (NSCLC) cells [113]. The cancer cell types included those sensitive (parental) and resistant to the anaplastic lymphoma kinase inhibitor alectinib. With two cell types, the replicator dynamics describing the change in frequencies of the parental, *q*, and resistant, $1-q$, cancer cell types in the population, become

8 $$\begin{aligned} \frac{\mathrm{d}q}{\mathrm{d}t}=q(1-q) \left( \left( a_{12}-a_{22}\right) (1-q)-(a_{21}-a_{11})\,q\right) , \end{aligned}$$

with a fitness matrix ${\mathbf {A}}=\left( a_{ij}\right) \!.$ Kaznatcheev et al. estimated the entries of the fitness matrix ${\mathbf {A}}$ in Eq. (8) from the growth data of a series of specifically designed in vitro experiments across four different environmental conditions corresponding to the presence or absence of targeted therapy and the presence and absence of cancer-associated fibroblasts [113]. They showed that the games played by the population in vitro produce two qualitatively different dynamics regimes, i.e., that the dynamics  (8) switch the type of game being played by the population in vitro from a game they term a ‘Deadlock game’ to a game they term a ‘Leader game’, based on the presence or absence of drug and/or fibroblasts.

While therapy optimization was not the goal of this study (in fact, therapy eventually failed for all considered cases), Kaznatcheev et al. provided the game assay as a method to estimate the entries in the fitness matrix from in vitro data [113]. This allows the physician to anticipate treatment-induced eco-evolutionary responses of cancer cells even before the treatment is applied in order to steer the eco-evolutionary dynamics of cancer cells during the course of the treatment [113, 164]. Subsequent work focused on quantifying competitive release in NSCLC [68] and extended the original game to a game with three types of cancer cells [34]. A similar method was used to observe host-parasite-like interactions between cancer cell types due to paracrine behaviors [142].

### Replicator Dynamics with Nonlinear Fitness Functions

Although the system studied by Kaznatcheev et al.  is well served by replicator dynamics with fitness given by the linear function $({\mathbf {A}}{\mathbf {q}})_i$, their method can also be used to estimate parameters for nonlinear fitness functions, i.e., a generalization of (7)

9 $$\begin{aligned} \frac{\mathrm{d}q_i}{\mathrm{d}t}= q_i \big (f_i({\mathbf {q}}) - \mathop {\sum }\limits _{j=1}^n q_j f_j({\mathbf {q}}) \big ), \end{aligned}$$

where the fitness functions $f_i$ are not necessarily linear [113].

This case of nonlinear fitness functions has generated extensive theoretical work in a public goods game, where cells can be producers (cooperators) or free-riders on shared resources produced by the others (defectors) [16]. The most relevant cases for cancer are the production of growth factors like vascular endothelial growth factors (VEGF) [12, 159], the production of hostile environments like acidity due to the Warburg effect [13, 14, 46], or the coupling of both [115]. Archetti et al. empirically estimated parameters of this nonlinear public goods game for neuroendocrine pancreatic cancer cells that produce insulin-like growth factor II, which supports proliferation and evasion of apoptosis [16, 17].

When the public good of VEGF production is coupled with the public good of tumor acidity, Kaznatcheev et al. showed that targeting the most common cancer cell type through MTD may lead to a worse long-term outcome for the patient than targeting less common types [115].

### Lotka–Volterra Models

As original replicator dynamics (7) assume that $\sum _{i=1} ^{n} q_i=1,$ extensions have been made to capture situations with a varying total population size. Such extensions involve fictitious free-space strategies [108, 192], but also more general dynamics [101, 123, 186]. A relatively large body of literature models interactions between cancer cells of different types and/or interactions between cancer cells and the environment through the Lotka–Volterra (LV) competition equations and their extensions [30, 55, 75, 206]. The LV equations were proposed separately by Lotka and Volterra to describe competition in one set of models and predator-prey dynamics in another one [120, 189]. Here, we restrict ourselves to the competition models.

While initially the LV dynamics described interactions between two species only, they can be expanded to model interactions of cancer cells of *n* types. Moreover, it is possible to convert the replicator dynamics for *n* types into the LV model with $n-1$ types and vice versa, by converting the fitness matrix ${\mathbf {A}}$ into the *competition matrix* of the LV model and maintaining the same stable equilibria (attractors). The proof of this ESS equivalence can be found in [100] and [37]. The attractors of the LV dynamics correspond to the attractors of the replicator dynamics (7) and may correspond to the ESSs of the matrix ${\mathbf {A}},$ as discussed before. For instance, the ESSs of the replicator dynamics model of mCRPC of You et al. in [199] are the same as the ESSs of the LV model in [206]. The LV model describes ecological dynamics (3), while the evolutionary dynamics are trivial as the resistance trait does not evolve and therefore corresponds to (5) with the right-hand side of each equation equal to 0. Alternatively, only the resistant cancer cell type may have evolving resistance, and hereby carrying a ‘hurdle of evolvability’ [151].

Stable polymorphic equilibria may exist within tumors [22, 52]. If the dynamics of the tumor can be described via Eq. (7) or other dynamics leading to ESSs, then these polymorphic equilibria will correspond to ESSs [187]. Furthermore, polymorphic stability in heterogeneous tumor cell populations has been shown to exist explicitly for some cancers [17, 73].

Likely, the most influential LV competition model of cancer dynamics is that of Zhang et al. [206]. This model has been derived from the replicator dynamics in [199], while preserving their ESSs. Subsequently, the model was expanded so that it allowed for modeling abiraterone acetate treatment (further referred to as “abiraterone”), assuming that this treatment, applied together with androgen deprivation therapy (ADT), decreased the carrying capacity of cancer cells producing testosterone. Moreover, under androgen deprivation, the carrying capacity of cancer cells dependent on testosterone was made a linear function of the density of the testosterone producing cancer cells. As such, the originally noncooperative game between the three cancer cell types includes also cooperative elements. The LV formulation has the advantage of including population dynamics providing a more realistic modeling framework. This is because treatment aims at decreasing tumor burden while keeping the proportion of treatment-resistant cancer cells low. Replicator dynamics models typically capture only the latter, unless they include birth–death processes. The LV competition model described in [206] formed the basis of the adaptive treatment protocol used in a successful clinical trial (NCT02415621) for metastatic Castrate-Resistant Prostate Cancer. In this trial, serum Prostate-Specific Antigen (PSA) is considered as a measure of tumor volume and used as the basis for response assessments. The patients enrolled in the trial received abiraterone at MTD until their initial PSA levels dropped to half and resumed only when the PSA returned to its initial value. In this way, patients had individual treatment regimens with varying length of cycles with and without treatment.

Zhang et al. achieved this by simulating SoC with MTD of abiraterone combined with ADT, using clinically motivated parameters, to show how SoC strongly selects for the testosterone-independent cancer cell type, due to *competitive release* [50, 204, 206]. This means that resistant cancer cells eventually outcompete other cells. The above described protocol for *adaptive therapy* assumes that, in the absence of treatment, resistant cells are less fit than sensitive cells [206] (standard assumption on the fitness costs of resistance in ecology [1, 171]). This assumption can, however, be relaxed, as shown in [188]. Both the simulated adaptive therapy and the clinical trial treatment regimen applied abiraterone together with ADT until the tumor volume dropped below half of its initial value, as indicated by the blood serum level of PSA. From that moment on, abiraterone was discontinued, until the tumor volume recovered to its initial level. Then, the cycle was repeated. This has two anticipated effects:

1. Cancer cells are not dominated by the drug-resistant cell type.
2. The cumulative drug dose is lower.

An interesting finding is that a lower initial proportion of sensitive cells leads to longer periods of time until the PSA reaches its initial level. Adaptive therapy also results in a gradual increase in the resistant cells from cycle to cycle, but this happens much slower than with the SoC.

In summary, Zhang et al. demonstrated that this adaptive therapy regimen leads to a longer time to progression (TTP) than SoC therapy under any initial conditions [206]. With their simple but effective approach, the adaptive therapy is not yet completely optimized. Instead, the conditions to pause and restore the abiraterone treatment are rules of thumb related to the current tumor volume. The corresponding clinical trial has shown that patients’ TTP increased remarkably with this regimen. Recent updates of this clinical trial (NCT02415621) are consistent with the initial findings [206, 207]. The adaptive therapy protocol prolonged TTP with less than half of the cumulative drug dose and appears to be successful for all patients that were initially responsive. Currently, the patients’ median TTP has nearly tripled. Conversely, most patients receiving the SoC have progressed.

Cunningham et al. adopted optimal control theory to optimize the abiraterone therapy from [206] with respect to different criteria, such as minimizing the variance of the total tumor burden [54]. This will be discussed in more detail in Sect. 3.

Meanwhile, West et al. investigated a *multi-drug* approach for mCRPC [196]. For simplicity, they limited themselves to a two-drug approach where the secondary drug is supposed to suppress the sub-population which is resistant to the primary drug. Accordingly, they considered the treatment with docetaxel (chemotherapy) and abiraterone, considering also a cell type which is resistant to both docetaxel and abiraterone. They conducted simulations parametrized on patients that progressed in the mentioned clinical trial by Zhang et al. (NCT02415621) and reached the conclusion that the administration of docetaxel together with abiraterone would have significantly increased TTP [207]. Based on the first Zhang’s trial, more trials on adaptive therapy have been initiated (e.g., in melanoma—NCT03543969, in thyroid cancer—NCT03630120, and also the second Zhang’s trial in mCRPC—NCT03511196).

There are other examples of game-theoretic models guiding clinical trials. For example, West et al. consider a trial on stage 2 and 3 estrogen receptor-positive breast cancer and treatment with an aromatase inhibitor and a PD-L1 checkpoint inhibitor combination, which attempts to lower a preoperative endocrine prognostic index (PEPI) that correlates with relapse-free survival [195]. They adopted a game with a $4 \times 4$ fitness matrix, which was then embedded in an ecological model of tumor population-growth dynamics. The resulting model predicts evolutionary and ecological dynamics that track changes in the PEPI score. By comparing different possible treatment regimens, they proposed a therapy plan with a one-month kick start with the immune checkpoint inhibitor followed by five months of continuous combination therapy as the most effective therapy choice. Current practice either uses the drugs in combination or just uses the aromatase inhibitor.

LV models can be extended to include other cells interacting with the cancer cells, such as T-cells (as predators), as shown in [30, 149]. Alternatively, one may be interested in the role of non-immune cells, such as cancer-associated fibroblasts that may inhibit or facilitate the fitness of all or just some types of cancer cells [113, 197]. The parameters of LV models can also be inferred directly from in vitro experiments following a procedure similar to the game assay [142].

### Spatial Game-Theoretic Models and Related Work

There is evidence that spatial interactions among cancer cells and/or interactions of cancer cells with their environment influence *intra-tumor heterogeneity*, the spatial properties of tumors, and patient prognosis [124].

In space, tumors can be viewed as complex evolving structures, consisting of cancer cells, normal cells, blood vasculature, inter-cellular spaces, and various nutrients, such as oxygen and glucose [80, 129]. Cancer cells, often of distinct types, compete for space and nutrients and engage in direct interactions. They both contribute toward and are affected by their microenvironments, within which they consume available resources, to proliferate and survive [66]. Within these neighborhoods, there are eco-evolutionary feedbacks where limiting resources impact the total abundance of cancer cells, and interactions between tumor cells influence the frequency of cell types. Moreover, spatially explicit data (e.g., biopsies, histological samples and magnetic resonance imaging (MRI)) are becoming more and more available [163, 193]. Pathologists often measure and score spatial distributions of cancer cell types, vasculature, immune cells, and other tumor properties [147, 208]. Also, cancer biologists increasingly recognize the ubiquity of spatial heterogeneity within tumors [32, 124, 170].

For these reasons, spatially explicit models have increased in popularity. However, one has to be careful in inferring and interpreting game parameters from measurements in spatially explicit systems [109, 110].

Spatially explicit EGT cancer models can take the form of *diffusion processes* framed as partial differential equations [180] or models can be *agent-based* [48, 121, 122]. In some special cases, it is possible to use analytic techniques to transform and solve spatially explicit EGT models in the same way as the implicit models we described above [110, 114, 137].

In graph-based models, the cancer cells may be represented on vertices of a network, such as Voronoi graphs [15], motivated by the claim that real biological tissues appear closest to those [53, 118]. Alternatively, individual cells may occupy a space on a spatial grid described as squares or hexagons [148, 175]. Agent-based models can also consider continuous space where the cancer cells are represented by continuously varying spatial coordinates in one, two or three dimensions, often extending the replicator dynamics (7) into spatially explicit scenarios [24, 74, 199, 200]. In this case, the interactions between the different cell types are typically more or less local and depend on how cells interact with each other, how much they can move, how far do density-dependent effects with neighbors extend, and/or how far to place a focal cell’s daughter cell.

For example, You et al. modeled the interaction of mCRPC cells under ADT as an evolutionary game with three types of cancer cells (cells requiring testosterone, cells producing testosterone as a public good, and cells independent of testosterone) [199]. A fitness matrix defined a focal cell’s probability of proliferating when interacting with other cells. The ESSs and transient dynamics of the non-spatial version of this game were compared to the transient dynamics and eco-evolutionary equilibria of a spatial variant of this game. The spatial version was an agent-based continuous-space model with a birth-death process. Only when interactions between cancer cells of the spatial model were global did the resulting evolutionary equilibria correspond to the ESSs of the original nonspatial game.

## Game Theory of Cancer Treatment

From a game-theoretic perspective, the physician is not a real player when the treatment protocol is decided *a priori*. This is the case for continuous MTD, metronomic therapies, and adaptive therapy when the therapy switching rules are decided beforehand. This was the case in the models introduced in Sect. 2.

Here, we consider the case where the physician becomes a true player in the game. When viewing cancer as an evolutionary game between the physician and the cancer cells, a natural question arises: Can we drive cancer into a stable state, corresponding to either a cure or a chronic disease, which is not too harmful for the patient and can be maintained at a stable tumor burden? This concept of stability corresponds to the Evolutionarily Stable Strategies introduced in Sect. 2. Alternatively, if cure or stable tumor burden cannot be achieved, a relevant question is whether we can maximally delay undesirable states (e.g., too high tumor burden or too high level of resistance), by more dynamical treatment protocols than currently used as SoC. To this aim, we introduce an objective function to be optimized by the physician, $Q\left( {\mathbf {U}}(\cdot ),{\mathbf {x}}(\cdot ),{\mathbf {m}}(\cdot )\right) ,$ which varies with ${\mathbf {m}}(\cdot ) {\mathop {=}\limits ^\mathrm{{def}}}\left[ {\mathbf {m}}(t)\right] _{t\in [0,T]},$ ${\mathbf {U}}(\cdot ) {\mathop {=}\limits ^\mathrm{{def}}}\left[ {\mathbf {U}}(t)\right] _{t\in [0,T]},$ ${\mathbf {x}}(\cdot ) {\mathop {=}\limits ^\mathrm{{def}}}\left[ {\mathbf {x}}(t)\right] _{t\in [0,T]}$. We can refer to this function as the *Quality of Life* function of the patient. The physician’s goal is to find the optimal ${\mathbf {m}}^*(\cdot )$ which optimizes such an objective, i.e., find

10 $$\begin{aligned} {\mathbf {m}}^*(\cdot )=\arg \mathop {\max }\limits _{{\mathbf {m}}(\cdot )} Q\left( {\mathbf {U}}(\cdot ),{\mathbf {x}}(\cdot ),{\mathbf {m}}(\cdot )\right) , \end{aligned}$$

where *Q* has been decided by the physician and patient *a priori*. In such a situation, cancer cells are playing an evolutionary game with each other and their eco-evolutionary dynamics can still be described by Eqs. (3) and (5). However, they become followers in a Stackelberg (i.e., leader-follower) game, with the physician as a rational leader [164]. Since the followers are evolutionary players, we call these types of games *Stackelberg evolutionary games* (SEGs), in accordance with recent research on this topic [156, 158]. It is noteworthy that the physician, as the only rational player in this SEG, can anticipate and steer the eco-evolutionary response of the cancer cells defined by (3)–(5), while the cancer cells can only adapt to the actions already taken by the physician. The theory of Stackelberg games was originally devised in economics to conceptualize interactions with an imbalance in control or power, e.g., the competition between a market leader and follower [27, 97]. Its extension into SEGs not only applies to cancer treatment, but also to other problems involving a rational player interacting with an evolutionary system such as pest management, fisheries management, or the control of infectious diseases [39, 40, 96, 158].

Here, we divide existing literature into two categories:

1. SEGs with cancer cells in eco-evolutionary equilibria: Here, it is assumed that an equilibrium ${\mathbf {x}}^*$ and ESS ${\mathbf {U}}^*$ is reached for any given choice of ${\mathbf {m}}.$ Under this condition, we look for a constant ${\mathbf {m}}$ that maximizes $Q({\mathbf {U}}^*(\mathbf{m} ),{\mathbf {x}}^*(\mathbf{m} ),{\mathbf {m}})$.
2. SEGs where the cancer cells are assumed to be in their transient phase, with their eco-evolutionary dynamics driven by Eqs. (3)–(5).

When it comes to the objective of the leader, we identify two important categories of literature:

1. SEGs where the leader aims at steering the cancer cells into their eco-evolutionary equilibria, assuming application of a constant dose once such an equilibrium is achieved. Here, the goal for the patient is not cure. The strategy becomes “treat to contain”, similarly to what happens with chronic diseases.
2. SEGs with different objectives for the leader, such as minimization of the tumor burden, minimization of its variance, or maximization of the TTP.

In Table 1, we summarize these options and indicate the sections where each is discussed.

**Table 1:** Instances of Stackelberg evolutionary games (SEGs) of cancer treatment considered in this review

		Physician	
		steering to $({\mathbf {x}}^*,{\mathbf {U}}^*)$	Another objective
Cancer dynamics	Transient at $({\mathbf {x}}^*,{\mathbf {U}}^*)$	Section 3.1	Section 3.2
		–	Section 3.3

### Physician Steering Cancer into an ESS

Most cancer biologists and many modelers see cancer as only transient dynamics with little focus on the idea of reaching an equilibrium $({\mathbf {U}}^*,{\mathbf {x}}^*)$ of its eco-evolutionary dynamics, and even fewer within an explicit game-theoretic setting. However, there is evidence that eco-evolutionary dynamics in cancer cells do have attractors whether reached or not [67, 83]. These works report that if these equilibria are reached, cancer can be contained with a constant dose of treatment, lower than the MTD. Martin et al. and Carrère suggested that reaching the ESSs of the cancer dynamics may be a successful strategy for keeping the patient with a metastatic cancer alive [45, 123]. Cunningham et al. focused on steering mCRPC into an eco-evolutionary equilibria, for the model from [206], where one competition coefficient was increased to a value above 1 [56]. This was based on a study, demonstrating that the competition coefficients among different cancer cells of different types may often be above 1 [73]. Cunningham et al. first adopted a numerical *optimal control approach*, with a forward-backward sweep method to steer mCRPC to a sustainable eco-evolutionary attractor [56]. While they showed that reaching such an attractor is feasible for most patients, they focused also on a rules of thumb approach to reach these attractors if they are unknown, without complicated optimization of the treatment protocols. They demonstrated that *dose titration*, i.e., gradual increase in treatment dose, can lead to a sustainable ESS.

### Physician Optimizing Objectives Other than Reaching the ESS, While Cancer Cells are in Their Transient Phase

Martin et al. were probably the first authors who applied optimal control in cancer treatment, with focus on various objectives for the physician [123]. They considered a population of drug-sensitive and drug-resistant cancer cells, where the goal was to slow the growth of drug-resistant cells, which also served to maximize patient survival time. Three types of tumor growth models were investigated: Gompertz, logistic, and exponential. For each model, they adopted an analytical optimal control approach to find feedback controls that specify the optimal tumor mass as a function of the size of the resistant sub-population [27, 33, 150]. With exponential and logistic tumor growth, the tumor burden during therapy had little impact on survival times. With Gompertzian tumor growth, therapies maintaining a large tumor burden doubled or even tripled patient survival time. A revolutionary finding of this paper was that maintaining a high tumor burden is optimal for Gompertzian tumor growth and close to optimal for exponential and logistic tumor growth. Hence, it is not necessary to know the precise growth characteristics of a tumor to schedule anticancer drugs. Their results also implied that trying to contain the tumor may be the best strategy for keeping patients alive. A growing literature using optimal control to design better treatment strategies has emerged as a follow-up to this work [5, 116, 138, 185].

Orlando et al. modeled cancer cells trading off resistance between two different drugs with the physician minimizing the tumor burden through optimal control theory [144]. They showed that a relatively static treatment using both drugs at equal levels is optimal when cancer cells benefit from specializing in response to a single drug rather than a generalist resistance strategy, while a more dynamic treatment with the concentration of drugs varying over time is more effective when the cancer cells adopt a generalist resistance strategy [144].

Carrère focused on in vitro tumors, consisting of cells that were sensitive or resistant to a certain drug [45]. The setting was similar to [123], but with parameters validated by an in vitro study [45]. They adopted optimal control theory and showed analytically that to reduce the tumor volume while preserving its heterogeneity, one needs to apply lower than the MTD treatment dose.

Warman et al. focused on a fitness matrix model of the vicious cycle of metastatic prostate cancer cells co-opting bone remodeling [192]. The authors introduced *fractionated follow-up therapy*—chemotherapy where dosage is administered initially in one solid block followed by alternating smaller doses and holidays—and showed that it is better than either a continuous application or a periodic one.

In [88], Gluzman et al. optimized treatment in a public goods model of interactions between glycolytic and acidic cells, introduced by Kaznatcheev et al. [115]. The total drug usage and time to recovery were optimized by solving the corresponding Hamilton–Jacobi–Bellman equation, similar to [56]. They concluded that the optimal treatment policies can significantly decrease the total amount of drugs prescribed, while also increasing the fraction of initial tumor states from which recovery is possible. This paper supports the claim that lower doses of treatment will be more effective for containing tumors than MTD.

Cunningham et al. optimized abiraterone treatment from [206] using boxed-constrained optimized [54]. They considered various objectives for the physician and show that minimization of the tumor volume variance, thus keeping the tumor burden as stable as possible, may be the best objective for keeping the patients from progressing while not applying too much drug.

Itik et al.  introduced a model describing competition between normal cells and tumor cells [105]. The model also includes the effects of the immune system. They proposed a linear time varying approximation technique to construct an optimal control strategy for the nonlinear system which is valid not only within small perturbations around the equilibrium point, but also for global dynamics of the system. The objective was to eliminate the tumor cells while minimizing the amount of drug. It should be noted, that as evolution of resistance is not included in the model, it is likely more relevant for treatment of early stage cancers, as opposed to advanced and metastatic cancers.

### Physician Optimizing Various Objectives, While Cancer Cells Dynamics are at ESS

**Fig. 1:**
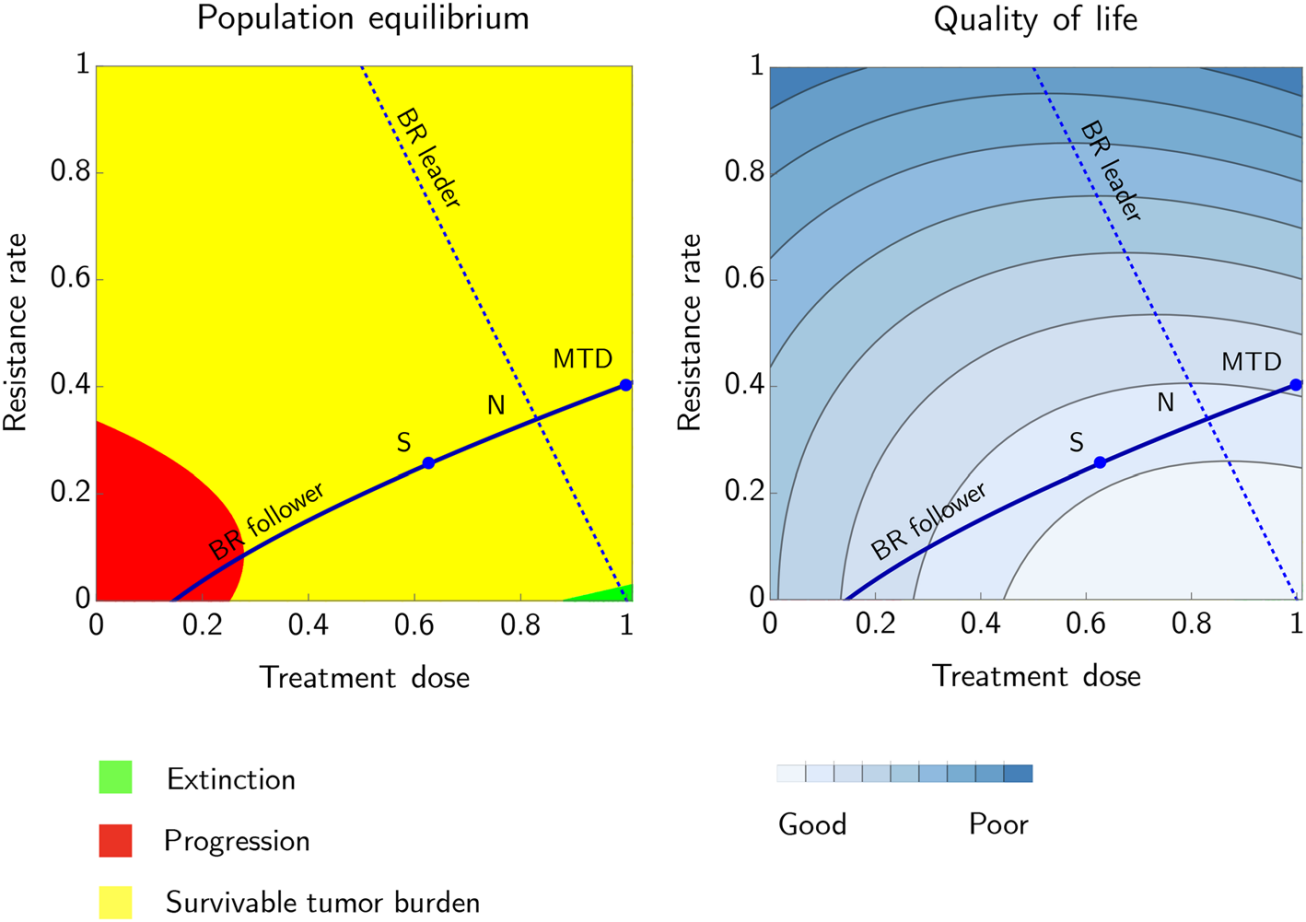
Illustration of the difference between Stackelberg equilibrium, Nash equilibrium, and Maximum Tolerable Dose in the cancer treatment game. The solid line represents the best response of cancer cells (followers) to any possible scalar treatment level $m\in [0,1]$, the dotted line the best response of the physician (leader) to any possible resistance level $u\in [0,1]$. The panel on the left shows the cancer cells population at equilibrium, while the panel on the right shows the quality of life of the patient for the same situation. In the green area, the population of cancer cells goes extinct, while in the red area it grows above the survival threshold of the patient and as such, it is incompatible with life. The yellow area represents the situation in-between, with different levels of quality of life. Three different outcomes of the game are presented: ‘MTD’ corresponds to the case where the physician plays a fixed Maximum Tolerable Dose strategy, ’N’ corresponds to adjusting the dose according to the resistance rate of cancer cells, until a Nash equilibrium is reached, and ’S’ corresponds to anticipating the cancer cells’ resistance strategy. Adapted from [157] (Color figure online)

Once the ecological equilibrium ${\mathbf {x}}^*$ and the ESS resistance strategies ${\mathbf {U}}^*$ are reached, a constant dose ${\mathbf {m}}^*$ can keep the cancer dynamics contained [156, 157, 165]. Finding such equilibria for cancer eco-evolutionary dynamics and ${\mathbf {m}}^*$ for maximizing the patient’s quality of life was the main goal of [156] and [158]. For monomorphic cancer cell populations, less treatment leads to a higher quality of life (Fig. 1; from [157]).

Their approach considered a monomorphic population of cancer cells, with evolving resistance as a scalar trait. However, the fact that MTD leads to an outcome which is not better and usually much worse in terms of quality of life than the Nash equilibrium, which is in turn not better and usually much worse than the Stackelberg equilibrium, can be generalized to the situation with vector-valued traits and to the case of a polymorphic population of sensitive and resistant cells.

## Clinical Relevance

Application of EGT principles in therapy, in order to anticipate and steer cancer eco-evolutionary response, is a powerful tool, but relies on our ability to estimate tumor size and composition prior to treatment. The intra-tumoral evolutionary process leads to sub-clonal diversification and generates the genetic and phenotypic intra-tumor heterogeneity, which determines the tumor composition and therefore the evolutionary state [124]. In order to optimize the model parameters, determined by the tumor composition, monitoring of the tumor’s behavior during therapy is required. At best, this encompasses continuous surveillance of the total number of tumor cells and their cell type composition. In clinic, the personalized therapeutic strategy then needs to be optimized after every measurement, i.e., after each clinical visit. Kaznatcheev et al. recently showed how to assess the game played by different cell types of non-small cell lung cancer cells in vitro [113]. This game changes in response to different treatment regimens. Due to the in vitro setup, the experiments could be monitored with relative ease by performing time-lapse microscopy. However, in a clinical setting the key constraint is the low amount of information available about intra-tumoral evolution and the speed of evolution during treatment. It is still challenging to identify, quantify and monitor the evolving strategy distribution in heterogeneous tumors. A sufficient technology for this is yet unavailable; however, several techniques can be proposed which we discuss in the following paragraphs.

Firstly, tissue biopsies of the primary tumor and of metastases can be sampled to reveal genetic and phenotypic differences between cancer cell types. Genetic differences are revealed by genome sequencing, while phenotypic heterogeneity is typically assessed with histology techniques and proteomics [32]. Nevertheless, to monitor the cancer cells’ response to treatment, tissues need to be isolated at the time of initial diagnosis as well as successively sampled throughout treatment. In the clinic, such repeated biopsies are not easily acceptable, due to their invasive nature and expense. Such is the case in taking biopsies of disseminated bone disease in mCRCP patients [72]. Furthermore, often only a fraction of the tumor is isolated, which does not represent the complete genomic and phenotypic landscape, and the detection of small lesions and deriving biopsies from them is a major challenge [104, 145].

Secondly, an alternative approach is based on liquid biopsies. They consist of several sources of tumor material including circulating tumor DNA (ctDNA) and circulating tumor cells (CTCs). The ctDNA is a DNA released by malignant cancer cells, with diagnostic genetic and epigenetic alterations. Several studies have shown that exome-wide analysis of ctDNA may contribute to monitoring the evolution of acquired drug resistance and track the outgrowth of resistant cell types [42, 135, 136, 143]. To be able to use the genotypic information obtained from ctDNA, we need to know the relationship between mutations and their phenotypic impact, i.e., the genotype-phenotype map [4, 141]. Predicting what genotypes will eventually evolve to drive phenotypic resistance remains a significant challenge [63].

From CTCs, besides genotypic information, phenotypic information about the strategy distribution can directly be obtained for use in the EGT models. CTCs represent intact, viable non-hematological cells with malignant features. The resistant CTC populations may be phenotypically distinct from their precursors in physical size, shape and surface marker expression. For instance, Tsao et al. detected tumor progression and proliferation of resistant melanoma cell types by observing surface marker up-regulation from CTCs [183]. They saw how a widening of the signal distribution detected by spectroscopy, reflected a more heterogeneous CTC population. In mCRPC, Zhang et al. detected testosterone producing cells by the presence of CTCs expressing CYP17A1, which is a key enzyme for androgen synthesis [206, 207]. Androgen receptors (AR) can also be detected and monitored in real time from mCRPC CTCs. The AR splice variant 7 was proved to be predictive of resistance to anti-AR treatment, such as ADT therapy and treatment with both abiraterone and enzalutamide [8, 131, 132, 166, 178]. Additionally, CTCs can be assayed for human epidermal growth factor receptor 2 (HER2) in breast cancer, which contributes to treatment resistance [51, 152]. This technique may also be applied to display the strategy distribution in other cancer types, when specific up-regulation or down-regulation of specific surface markers in resistant cell types occurs.

Taking liquid biopsies and isolating CTCs has advantages over conventional tissue biopsies since they are less invasive to the patient. Additionally, it may reflect the heterogeneity of the tumor more appropriately and it allows continuously monitoring of a patient’s tumor composition [119]. Nevertheless, the liquid biopsies provide neither spatial information nor information on the composition of individual metastatic lesions, since the primary tumor and its metastases are not measured individually. Accordingly, liquid biopsies may contain a mixture of tumor cells originating from multiple independent lesions. Analyses of primary and disseminated tumor cells show large differences in genetic variation [167], and CTCs are unlikely to represent the full spectrum of mutations and differences in protein expression in tumor lesions since CTC biopsies might only show the ‘tip of the iceberg’ [205]. While it is better to have this aggregated information as a proxy for the cancer’s evolutionary dynamics than no information at all, the information found this way can be used to measure the evolutionary states of different metastatic lesions if multiple metastatic lesions located at different sites shed CTCs homogeneously or if the variation in the composition of these lesions is low. This is shown in BRAF status concordance in primary and metastatic melanoma [35, 36] and colorectal carcinoma and KRAS mutation status in colorectal adenocarcinoma [60]. Alternatively, one needs to identify the tissue of origin of CTCs by using expression profiling of organ-specific metastatic features. Studies have shown that certain methylation patterns are tissue specific, which may serve to determine the source of tumor cells or ctDNA [117, 169].

Thirdly, another approach is blood sampling to measure blood serum markers. These are biomarkers produced by specific tumor cell types. In studies by Zhang et al., prostate cancer volume is determined by assessing PSA levels in the blood, while in the latter research testosterone blood levels under androgen deprivation are measured [206, 207]. The testosterone levels are used as a proxy for the amount of testosterone-producing cancer cells. Nevertheless, whether each tumor cell produces the same amount of PSA might depend on the sensitivity of the tumor cells to androgen stimulation for the expression of PSA. Some cell types have been shown to lose sensitivity to androgen and produce even more PSA than androgen sensitive cell types [59, 106]. This feature that differs between prostate cancer cell types might provide ways to measure androgen-independent and dependent types of cancer cells. However, this is again aggregated information combining all metastatic lesions. A study in melanoma showed a higher expression of BRAF (V600E) oncoprotein in vemurafenib-resistant tumor cells compared to sensitive cells. This difference might be used for parameterizing EGT models of melanoma [174]. For other cancer types investigated in adaptive therapy studies, there is a lack of reliable biomarkers presented yet.

Fourthly, modern imaging techniques are another emerging approach for gaining tumor and intra-tumoral metrics. Imaging can provide a holistic view of the entire tumor and since it is noninvasive, it is suitable for repeated monitoring. Magnetic resonance imaging (MRI) and computed tomography (CT) can be used to track spatial and temporal patterns of heterogeneity. For example, these techniques may reveal tumor habitats such as necrosis, hypoxia and vascular permeability. Such habitats may select for different cells with varying levels of responsiveness to therapy [176, 177].

Radiomics provide images of tumor habitats which seek correlations between cell phenotypes and their visual appearance. Quantitative imaging features can include shape, edge to volume ratio, texture or tissue environment. Such features can be built into predictive models relating image features to tumor cell types [3, 81]. It has already been demonstrated in studies of patients with glioblastoma multiforme that differences in cancer cell protein expression within a tumor correlate with regions of varying contrast-enhancement from MRI images [62, 99]. However, before quantitative imaging features can be used for clinical monitoring of cancer cell strategies, it needs to be ensured that specific imaging features can be linked to the underlying composition of cell types that differ in their response to treatment.

Positron Emission Tomography (PET), which can be performed along with CT or MRI scans, provides additional anatomic and spatial information. PET scans can show differential amounts and patterns of uptake of radiotracers by cells within a tumor. This might provide the ability to label and quantify the resistant as well as the sensitive cells. For example, the variability of tumor glycolytic metabolism within the same lesion can be assessed with the use of 2-flouro-2-deoxy-D-glucose F 18 ([18F]-FDG) PET imaging [28]. Uptake patterns influence, thus patients’ outcome and thus provide insights into the prevalence of resistant cancer cells within the tumor. Additionally, PET imaging using fluorodihydrotestosterone F 18 ([18F]-FDHT) permits labelling and detection of androgen receptors [202]. Accordingly, a combination of [18F]-FDG and [18F]-FDHT PET imaging can identify AR positive and negative lesions, and therefore the ability to discriminate sensitive and resistant prostate cancer cell types [71]. A radiotracer to label prostate-specific membrane antibody (PSMA), a cell surface protein with high expression in prostate cancer cells, is also available for PET imaging. PSMA is expressed on nearly all prostate cancer cells, and therefore accessible to labelling [126]. Furthermore, it is under research whether the radiotracer N-succinimidyl-4-[18F]fluorobenzoate ([18F]-SFB) is suitable for labeling HER2 overexpressing cells in breast cancer [198].

Modern imaging techniques may hold more promise than tissue and liquid biopsies. This is because it can reveal relevant information about both the location of the lesions and the tumor cell types within these lesions, to reveal both tumor eco-evolutionary dynamics and spatial characteristics. Furthermore, it is noninvasive and overcomes sampling errors of biopsies. In particular, we propose PET imaging because it can provide insight in both total tumor mass and the tumor’s cell type composition. Therefore, the discovery of radiotracers, which are able to classify different tumor cell types, is of uppermost clinical importance. Nevertheless, current modern imaging studies mostly focus on how tumor metrics and not cell type composition can be used as a prognostic marker for overall survival, malignancy or therapy response. For example, Aerts et al. used radiomic data from CT images of patients with early stage NSCLC and used a response phenotype that can predict a patient’s sensitivity toward Gefitinib therapy [2]. In order to parameterize the EGT models, all different tumor cell types in a tumor need to be identified and monitored.

It may be worthwhile to use newly developed techniques such as organoids [155, 184] and xenografts [41] to measure cell type compositions and protein expression to monitor tumor evolution and improve our understanding of the eco-evolutionary dynamics. Early preclinical in vivo studies of adaptive therapy included ovarian cancer cell line xenografts treated with carboplatin, and MDA-MB-231/luc triple-negative and MCF7 ER+ breast cancer cell lines treated with paclitaxel. In all cases, adaptive therapy could stabilize tumor volume, though the underlying sub-populations were not explicitly measured [67, 83]. In both of these studies, once initial tumor volume control was achieved, it could be maintained with constant or even progressively smaller drug doses, suggestive of stable eco-evolutionary equilibria.

Once patient-specific data of tumor cell types are available and monitored, it can be used to parameterize and optimize the EGT models to guide adaptive therapy protocols [98]. Subsequent measurements to inform patient specific parameters would then greatly improve modeling and predictions regarding tumor characteristics [206, 207]. After every measurement, the optimal next step in the adaptive therapeutic protocol could be calculated and used to stabilize the tumor burden, or may even be steered to create a pathway toward cure. To compare different mathematical models and seek the optimal cancer treatment, an optimal control theory approach may suffice [7, 47, 54, 57, 133, 134, 160]. Additionally, model predictive control (MPC) can use real-time monitored data to update the optimal cancer treatment. MPC involves model-based control techniques which can update the model and the optimal treatment schedule with each new clinical measure [134].

Critically, a model for tumor treatment can only be as effective as its associated empirical methods allow, i.e., in order to parameterize and validate it. Data may be retrospective (histologies, radiographies, biopsies, etc.) as well as derived from mouse or cell culture studies. For mapping genotypic or phenotypic data to treatment strategies, traditional statistical approaches can be used, but opportunities for machine learning and/or artificial intelligence are evident [43, 107]. Furthermore, the in vitro and in vivo competition assay has been shown to be well suited to feed EGT models. Such experiments have already shown that the success of cancer lineages depends on its frequency and the frequency of all other lineages with other strategies [23, 102, 103]. General models, when augmented by measurements, will permit EGT to inform clinical practice [89].

## Discussion

We have reviewed the application of EGT in modeling tumor progression with and without treatment. When considering treatment models, we made a distinction between those with *a priori* defined treatment or no treatment vs. those where the physician enters the game and actively adjusts treatment strategies during the course of the treatment in response to the metrics of the cancer’s eco-evolutionary state.

We considered evolutionary approaches to treatment and anticipated increased life expectancy from evolutionary therapy as compared to the traditional therapy, when resistant types are either pre-existing or evolve in response to therapy.

The biggest obstacle to applying EGT treatment methods to clinics remains the difficulty of estimating the tumor composition which currently can be done only for some types of cancers. Therapy for such cancers is particularly suited for our approach. They have discrete cell types and can therefore be understood via simpler EGT models. We reviewed some approaches for estimating the tumor composition in Sect. 4.

There is a clear gap between the complexity of the models that we introduced by (3) and (5), with a resistance matrix ${\mathbf {U}}$, and existing models, where either scalar or vector-valued traits are considered. We could find no research where one actually considers the resistance level of each known cancer type to each possible treatment. In all research we reviewed, resistance was either a single evolving trait of a monomorphic cancer population, a strategy within a polymorphic cancer population with one treatment, or multiple strategies of a polymorphic population with multiple treatments, where the resistance to these treatments does not evolve according to (5) but represents discrete and fixed strategies. The effects of these modeling assumptions (monomorphic or polymorphic population) and how they impact the superiority of adaptive therapy over continuous therapy with MTD have been recently investigated by Pressley et al., where time to progression for monomorphic and polymorphic models was compared between adaptive therapy and MTD [151]. The most general form of the cancer model, given by (3) and (5), has recently been used by Reed et al. to model pediatric sarcomas, where tumor growth is suppressed by multiple drugs, toward which resistance is evolving (e.g., vinorelbine, dactinomycin, cyclophosphamide) [153].

Most commonly used replicator dynamics and LV equations describe only one of the Eqs. (3) and (5). There is a rich theory for both, presumably as these models are simpler than the most general ones.

Some topics are not addressed in this paper that may become relevant for the future use of game theory for cancer and its treatment. For example, we did not specify whether the cancer cells’ types correspond to genetic or non-genetic traits (e.g., epigenetics; [161]). Generally, we believe that this does not influence the conceptualization of the game-theoretic models. Furthermore, whether strategies are genetic, epigenetic or phenotypically plastic will, at times, influence evolutionary speed. It may be that the tumor micro-environment can influence the epigenetics of a cell and thereby change its type, while this would not be the case if the type is genetically determined. Future models may need to pay close attention to the role of the micro-environment on the capacity for cancer cells to switch strategies. This switching may also happen in cancer stem cells (CSCs) and have consequences for tumor heterogeneity and the composition of cancer cell types. This may be interesting to study in the context of EGT modeling and cancer therapy.

Many common cancer types are shown to be propagated by small populations of CSCs. Genetic and epigenetic alterations can lead to CSCs emerging from non-stem cells endowed with stem cell properties. Therefore, stem cell identity may not be strictly a property of that cell, but may also depend on extrinsic cues provided by the adjacent cells and microenvironment. If stemness is not an intrinsic property, the malignant cells will regenerate new cancer stem cells, even if those with stem-like properties have been eliminated. Accordingly, the stem state of a cell is a dual phenotype. Therefore, in modeling CSCs a choice must be made on whether stemness is an intrinsic property or whether cell type switching takes place or not. Analysis of the evolution of stemness can help to identify whether these different types of stemness evolve according to different selective pressures, such as tissue maintenance and repair. This phenomenon also poses the question as to how non-genetically encoded plasticity will affect EGT modeling.

To conclude, many game theoretical models hypothesize on cancer behavior that yet has to be validated with real data [29, 31, 151, 188]. Close communication and collaboration between theoretical and empirical scientists will be of the utmost importance in advancing evolutionary therapies based on evolutionary game theory, to improve treatment results and patients’ quality of life.

## Abbreviations

ADT: Androgen deprivation therapy
AR: Androgen receptor
CSC: Cancer stem cell
CT: Computed tomography
CTC: Circulating tumor cell
ctDNA: Circulating tumor DNA
EGT: Evolutionary game theory
ER+: Estrogen receptor positive
ESS: Evolutionarily stable strategy
HER2: Human epidermal growth factor receptor 2
LV: Lotka–Volterra
mCRPC: Metastatic castrate-resistant prostate cancer
MPC: Model predictive control
MRI: Magnetic resonance imaging
MTD: Maximum tolerable dose
NSCLC: Non-small cell lung cancer
PEPI: Preoperative endocrine prognostic index
PET: Positron emission tomography
PSA: Prostate-specific antigen
PSMA: Prostate-specific membrane antibody
SEG: Stackelberg evolutionary game
SoC: Standard of care
TTP: Time to progression
VEGF: Vascular endothelial growth factors
